# Utilization and outcomes of single-troponin discharge from the emergency department in a low-risk population

**DOI:** 10.21542/gcsp.2025.8

**Published:** 2025-02-28

**Authors:** Zhengqiu Zhou, Kory Heier, Jeffrey F. Spindel, Austin Harris, Emily Slade, Weston McCowan, Dealla Samadi, Joshua Kim, James D. Moore, Seth T. Stearley, Vedant Gupta

**Affiliations:** 1 Department of Emergency Medicine, HCA Florida North Florida, USA; 2 Department of Graduate Medical Education, Gainesville, FL, USA; 3 University of Central Florida/HCA Florida Healthcare, Gainesville, FL, USA; 4 Department of Biostatistics, University of Kentucky, Kentucky, USA; 5 Division of Cardiovascular Medicine, University of Kentucky Gill Heart and Vascular Institute, Kentucky, USA; 6 Division of General Internal Medicine, University of Kentucky, Kentucky, USA; 7 Department of Cardiology, St. Joseph Hospital & Medical Center, Creighton University School of Medicine, Phoenix, AZ, USA; 8 Department of Emergency Medicine, College of Medicine University of Kentucky, USA

## Abstract

**Objectives:** There is emerging data supporting rapid triage of low-risk chest pain patients to help facilitate Emergency Department (ED) throughput. We assessed an algorithm for accelerated rule-out of acute coronary syndrome in low-risk patients with an undetectable initial high sensitivity cardiac troponin (hs-cTn assays; Roche Diagnostics) in a real-world clinical setting.

**Methods:** All adults presenting with chief complaint of non-traumatic chest pain to our tertiary care ED with HEART score ≤3 with at least one hs-cTn and EKG obtained were included in our study. Data and outcomes were compared three months before and after implementation of our updated Acute Chest Pain Optimal Care Pathway that allowed discharge of patients with an initial troponin below detectable range (<6ng/L). The primary outcome was acute coronary syndrome at presentation or major adverse cardiovascular events within 30 days. Secondary outcomes included ED length of stay, cardiology consultations, and hospital admissions.

**Results:** Of the 229 patients fitting our criteria with a low-risk heart score (≤3) and undetectable initial troponin, zero patients were diagnosed with ACS and one major adverse cardiac event occurred post-implementation (0.8%). There were no significant differences in median ED length of stay pre- (4.5 h, IQR 3.8–6.6) versus post-implementation (4.6 h, IQR 3.5–5.7; *p* = 0.448), number of cardiology consults (*p* = 0.305) and hospital admissions (*p* = 0.261). However, protocol adherence was poor (50%). In a subgroup analysis post-implementation, patients whose care aligned with the algorithm (*n* = 64, 50.0%) had significantly shorter ED stays (median 4.1 h, IQR 3.3-4.9 vs 5.2 h, IQR 4.4-6.9, *p*¡0.001), fewer admissions (*p* = 0.001) but not fewer cardiology consults (*p* = 0.440).

**Conclusions:** Our study supports current data regarding the safety of accelerated rule out strategy with hs-cTn. However, our conclusions are limited due to its small sample size and retrospective design.

## Introduction

Chest pain is among the most frequent causes of emergency department (ED) presentations in the United States^1^. It is imperative to quickly and accurately distinguish life-threatening causes of chest pain, such as acute myocardial infarction (AMI), from more benign etiologies. Traditional diagnostic algorithms relied on Troponin I or T values with 1-2 h repeat lab draws. However, with the development of high-sensitivity cardiac troponin (hs-cTn) assays, these newer assays have demonstrated superiority in randomized controlled trials and have become the standard of care^2–6^. Furthermore, in low-risk patients, studies have demonstrated that an initial troponin level below the limit of quantification (LOQ) effectively rules out acute myocardial infarction (AMI) in the vast majority of chest pain patients^5–7^. This is currently supported by the European Society of Cardiology Guidelines^8–10^. Some authors have even suggested that a pre-hospital AMI rule-out could be safe and cost effective^11^.

**Figure 1.**
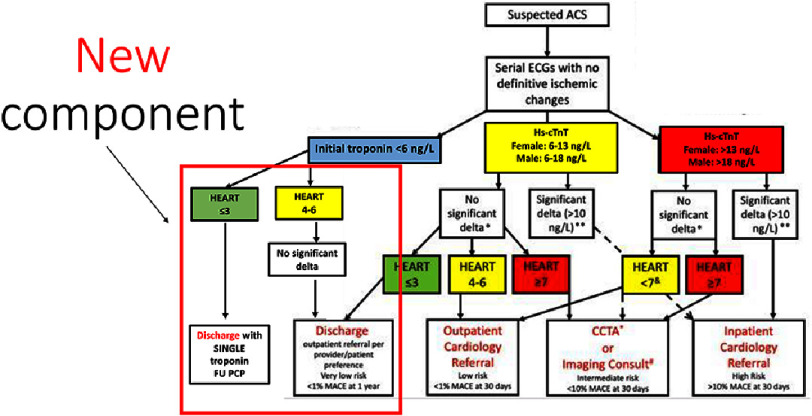
Institution-based protocol termed “Acute Chest Pain Optimal Care Pathway” highlighting updated component for diagnosis or rule-out of acute myocardial infarction. ACS- acute coronary syndrome; MACE – major adverse cardiac event; <6ng/L, 6-13 ng/L, 6-18 ng/L, >13 ng/L, >18 ng/L refers to Roche 5th generation hs-cTn values, CCTA- coronary CT angiogram. Gender specific cut off for >99th percentile is >13ng/L for females and 18 ng/L for males. * refers to 0 and 2 h hs-cTn difference <10 ng/L. ** refers to 0 and 2 h hs-cTn difference >10 ng/L. Figure modified from Zhou et al. ^12^.

In 2018, our tertiary care center developed and implemented a novel chest pain evaluation protocol, termed the ”Acute Chest Pain Optimal Care Pathway”. This pathway combines the use of hs-cTn assays (Roche Diagnostics, Indianapolis, IN) with the HEART score for risk stratification. The implementation of this protocol has resulted in significant improvements in various ED throughput metrics, including reduced length of stay (LOS), improved cardiology consultation efficiency, and decreased admission rates^12^. These benefits were seen while maintaining a low major adverse cardiac event (MACE) rate at 30 days after discharge. However, based on the more recent findings that AMI can be safely ruled out in low risk patients without the need for serial troponin measurements, we hoped that we can further improve our ED throughput metrics. Hence, in 2021, we revised our chest pain algorithm to incorporate an accelerated rule-out of acute coronary syndrome (ACS) and discharge pathway for chest pain patients who are classified as low cardiac risk - defined by a HEART score of ≤3, with a single below LOQ (troponin <6 ng/L; Figure 1). In this study, conducted a retrospective analysis to evaluate the compliance with and outcomes of our updated protocol.

## Methods

In a multi-disciplinary collaboration, the ED implemented an updated “Acute Chest Pain Optimal Care Pathway” on October 1, 2021. Prior to this implementation, a lecture was delivered on the morning of September 30, 2021, during the resident conference. The lecture included a brief presentation of current evidence on safety of discharge with single undetectable troponin in ACS rule-out as rationale for protocol update and then explained the revised protocol. Attendees were given opportunity to ask any questions regarding the changes. A follow-up email was sent to all ED providers and nursing staff afternoon of September 30, 2021, to inform them of the protocol update as not all providers will have attended resident conference. Laminated copies of the updated protocol were placed in physician and provider computing areas throughout the ED on morning of October 1. Since the implementation of the updated protocol, multiple email and text message reminders have been periodically sent to encourage the documentation of the HEART score in patients presenting with chest pain. The HEART score, defined by Six et al.,^13^ has been validated when utilizing troponin I assays in both a multinational validation study and a metanalysis^14,15^.

We conducted a retrospective analysis of all patients presenting to the ED with non-traumatic chest pain that had at minimum one troponin lab and one EKG at the time of their ED presentation 3-month period prior and 3-months after implementation of the updated algorithm on October 21st, 2021. Patients were included in the retrospective analysis if they presented with non-traumatic chest pain and were older than 18 years of age. Only patients in the “low risk” category (HEART score ≤3) and initial troponin undetectable high-sensitivity cardiac troponin assay (<6ng/ml) were included in our analysis. Electronic medical records were manually reviewed for demographics, clinical risk factors, lab values, EKG findings, diagnosis, procedures and clinical outcomes at 30 days after presentation. Physicians on study team manually reviewed and extracted data from hospital charts and any other records available through shared records from other local hospital systems. A single cardiology-trained physician reviewed all EKGS and documented the “EKG” portion of the HEART score. HEART score was only seldomly documented (8.4%) in electronic medical record (EMR) documentation; therefore, it was retrospectively calculated based on chart review. Only retrospectively calculated HEART score was used in our analysis to maintain consistency across patients. The specific criteria for HEART score determination is provided in the supplemental methods.

The primary outcome was ACS at presentation and MACE within 30 days. MACE was defined as any incidence of AMI, stroke, hospitalization for heart failure, or cardiac death that did not occur at initial visit. AMI was defined by the Fourth Universal Definition of Myocardial Infarction^16^. Stroke or hospitalization for heart failure were identified from the admission diagnoses. Cardiac death was defined as death from a cardiac cause listed on the hospital death summary or obituary. Secondary outcomes were length of stay in the ED (time), cardiology consultation (yes/no), and hospital admission (yes/no). All data were manually obtained via chart review by trained research staff.

Summary statistics were generated for variables of interest stratified by before and after implementation and by one vs two troponin labs in the after-implementation group. Categorical variables were reported using frequencies and column percentages (%). Continuous variables were assessed for normality using the Shapiro–Wilk normality test along with histograms. Continuous variables were reported using medians, and interquartile range (IQR). *P*-values for categorical variables were generated using either Pearson’s chi-square tests or Fisher’s exact tests. *P*-values for continuous variables were calculated using the Wilcoxon rank sum test. A multivariable analysis was performed using a negative binomial model to examine the relationship between ED LOS and protocol adherence after adjusting for the individual components of HEART score. To investigate temporal trends post-implementation, a logistic regression was used to examine the association between protocol adherence and week the patient presented to the ED.

For all analyses, patients with missing data were excluded. All statistical tests were two-sided and statistical significance was defined as *p*-value ≤0.05. All analyses were done in R programming language, version 4.1.1 (R Foundation for Statistical Computing, Vienna, Austria). This study was reviewed and approved by the University of Kentucky IRB (#77310).

## Results

### Characteristics of study group

After excluding cases of traumatic chest pain and those under age of 18, we identified 1,073 patients with “non-traumatic chest pain” that had at least one troponin lab and EKG at index hospital visit. Of these, 507 had complete data and 229 met low risk criteria defined as HEART score less than or equal to 3 and initial troponin <6 ng/L. This encompassed 101 patients before and 128 patients after algorithm changes that were included in our final analysis. Age, sex, race, and past medical history were similar between the two groups (Table 1). There were no ACS events in any patient before implementation or after updated algorithm implementation.

**Table 1:** Patient demographics and clinical characteristics.

Characteristic	Overall *N* = 229	Before algorithm implementation *N* = 101	After algorithm implementation *N* = 128	*p*-value
Age				0.105
Median	36.0	37.0	34.0	
IQR	[28.0, 45.0]	[28.0, 47.0]	[28.0, 43.3]	
Sex				0.500
Female	151 (65.9%)	69 (68.3%)	82 (64.1%)	
Male	78 (34.1%)	32 (31.7%)	46 (35.9%)	
Race				0.227
Asian	6 (2.6%)	3 (3.0%)	3 (2.3%)	
Black or African American	36 (15.7%)	13 (12.9%)	23 (18.0%)	
Hispanic	23 (10.0%)	9 (8.9%)	14 (10.9%)	
Native American or Alaskan	1 (0.4%)	0 (0.0%)	1 (0.8%)	
Hawaiian or Pacific Islander	1 (0.4%)	0 (0.0%)	1 (0.8%)	
Other	1 (0.4%)	0 (0.0%)	1 (0.8%)	
Unknown	4 (1.7%)	4 (4.0%)	0 (0.0%)	
White, non-Hispanic	157 (68.6%)	72 (71.3%)	85 (66.4%)	
Atherosclerosis				0.036
Absent	223 (97.4%)	101 (100.0%)	122 (95.3%)	
Present	6 (2.6%)	0 (0.0%)	6 (4.7%)	
Hypertension				0.275
Absent	183 (79.9%)	84 (83.2%)	99 (77.3%)	
Present	46 (20.1%)	17 (16.8%)	29 (22.7%)	
Hyperlipidemia				>0.999
Absent	218 (95.2%)	96 (95.0%)	122 (95.3%)	
Present	11 (4.8%)	5 (5.0%)	6 (4.7%)	
Diabetes				0.308
Absent	211 (92.1%)	91 (90.1%)	120 (93.8%)	
Present	18 (7.9%)	10 (9.9%)	8 (6.3%)	
Obesity				0.833
Absent	132 (57.6%)	59 (58.4%)	73 (57.0%)	
Present	97 (42.4%)	42 (41.6%)	55 (43.0%)	
Family History of ACS				0.805
Absent	192 (83.8%)	84 (83.2%)	108 (84.4%)	
Present	37 (16.2%)	17 (16.8%)	20 (15.6%)	
Tobacco				0.104
Absent	175 (76.4%)	72 (71.3%)	103 (80.5%)	
Present	54 (23.6%)	29 (28.7%)	25 (19.5%)	

**Notes.**

*P*-values are based on Chi-square or Fisher’s exact tests for categorical variables and Wilcoxon rank sum tests for continuous variables.

Abbreviations IQR interquartile range ACS acute coronary syndrome

### Before and after implementation

We compared the number of troponin labs drawn, ED LOS, cardiology consults, admissions, and MACE events before and after updated pathway implementation (Table 2). The proportion of patients having one troponin drawn (vs. two) did not significantly differ before and after intervention (before  =  49.5% vs after 50.0%, *p* = 0.941). The median ED length of stay prior to implementation was 4.5 h, median ED length of stay after implementation was 4.6 h (*p* = 0.448). Additionally, there was no significant difference between the proportion of patients receiving cardiology consults (before = 2.0% vs after = 5.5%, *p* = 0.305), or the proportion of patients being admitted (before = 7.9% vs after = 12.5%, *p* = 0.261).

**Table 2:** Clinical care and outcomes before and after implementation of the updated Acute Chest Pain Optimal Care Pathway.

	Overall *N* = 229	Before Algorithm Implementation *N* = 101	After Algorithm Implementation *N* = 128	*p*-value
# of Troponin Labs				0.941
One	114 (49.8%)	50 (49.5%)	64 (50.0%)	
Two	115 (50.2%)	51 (50.5%)	64 (50.0%)	
ED LOS (hours)				0.448
Median [IQR]	4.6 [3.6, 6.1]	4.5 [3.8, 6.6]	4.6 [3.5, 5.7]	
Unknown, N (%)	20 (8.7%)	11 (10.9%)	9 (7.0%)	
Cardiology Consult				0.305
No	220 (96.1%)	99 (98.0%)	121 (94.5%)	
Yes	9 (3.9%)	2 (2.0%)	7 (5.5%)	
Admitted				0.261
No	205 (89.5%)	93 (92.1%)	112 (87.5%)	
Yes	24 (10.5%)	8 (7.9%)	16 (12.5%)	
MACE within 30 days				>0.999
No MACE	101 (100.0%)	127 (99.2%)	
MACE	1 (0.4%)	0 (0.0%)	1 (0.8%)	

**Notes.**

*P*-values are based on Chi-square or Fisher’s exact tests for categorical variables and Wilcoxon rank sum tests for continuous variables.

Abbreviations MACE major adverse cardiovascular event ED emergency department LOS length of stay IQR interquartile range

### Post implementation

**Figure 2.**
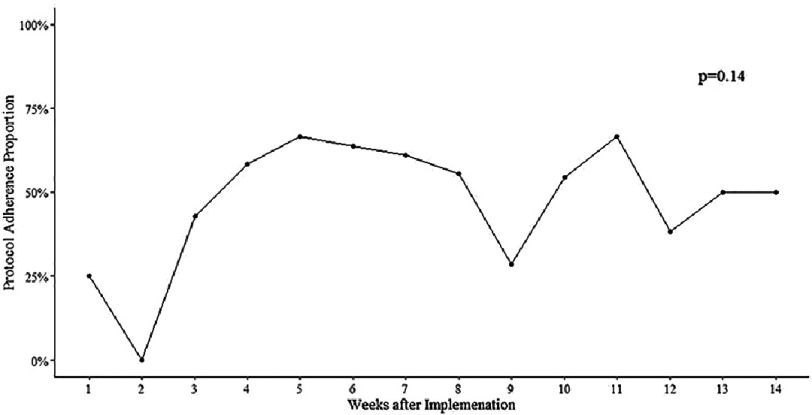
Line graph showing proportion of patients with protocol adherence over the 3 months period after implementation of the updated “Acute Chest pain Optimal Care Pathway”. Protocol adherence proportion was calculated as percentage of patients with initial troponin <6ng/L and HEART score ≤3 that did not get a second troponin drawn.

We then further conducted subgroup analysis of the 128 patients treated after pathway implementation that had HEART score ≤3 to evaluate if the pathway was followed. Protocol compliance was poor. We found that of the 128 patients that had initial undetectable troponin AND had HEART score ≤3, a second troponin was drawn in 64 patients (50.0%) despite the algorithm noting that only single troponin is necessary to rule out ACS. There was no significant difference between time after implementation and protocol adherence (*p* = 0.14, Figure 2).

In the 64 patients with a single undetectable troponin lab (50.0%), there was a significant difference in admission rate (one troponin  =  3.1% vs two troponins  =  21.9%, *p* = 0.001) and ED LOS (median hours [IQR] for one troponin  =  4.1 [3.3, 4.9] vs two troponins  =  5.2 [4.4, 6.9], *p*¡0.001, Table 3). The rate of cardiology consults, although ∼60% less in the single troponin group, was not significantly different than that in the two troponins group (one troponin  =  3.1% vs two troponins  =  7.8%, *p* = 0.440, Table 3).

**Table 3:** Clinical care and outcomes after implementation of the updated Acute Chest Pain Optimal Care Pathway, stratified by receipt of one *vs.* two troponin labs.

Characteristic	Overall *N* = 128	One troponin *N* = 64	Two troponin *N* = 64	*p*-value
ED LOS (hours)				<0.001
Median [IQR]	4.6 [3.5, 5.7]	4.1 [3.3, 4.9]	5.2 [4.4, 6.9]	
Unknown, N (%)	9 (7.0%)	6 (9.4%)	3 (4.7%)	
Cardiology Consult				0.440
No	121 (94.5%)	62 (96.9%)	59 (92.2%)	
Yes	7 (5.5%)	2 (3.1%)	5 (7.8%)	
Admitted				0.001
No	112 (87.5%)	62 (96.9%)	50 (78.1%)	
Yes	16 (12.5%)	2 (3.1%)	14 (21.9%)	
MACE Event				>0.999
No MACE	127 (99.2%)	63 (98.4%)	64 (100.0%)	
MACE	1 (0.8%)	1 (1.6%)	0 (0.0%)	

**Notes.**

*P*-values are based on Chi-square or Fisher’s exact tests for categorical variables and Wilcoxon rank sum tests for continuous variables.

Abbreviations MACE major adverse cardiovascular event ED emergency department LOS length of stay IQR interquartile range

We further conducted a multivariable analysis using a negative binomial model for ED LOS. The ED LOS was still significantly lower for one troponin vs two troponins while accounting for the “history”, “ECG”, “age”, and “risk factor” components of the HEART score in the multivariable analysis (*p* = 0.014). The multivariable analysis found that, after adjusting for the previously mentioned variables, those who had one troponin drawn had a 27% shorter ED length of stay than those who had two troponin labs drawn.

### MACE

There was no case of MACE prior to implementation and a single MACE event occurred in the post-implementation group. The MACE occurred in a patient that was initially discharged with a single undetectable troponin. The patient presented a second time for recurrence of chest pain within 30 days and had troponin elevation, was diagnosed with stress cardiomyopathy (nonobstructive coronaries on coronary CTA and a characteristic echocardiographic pattern), and admitted to the hospital for acute heart failure. No additional statistical analysis was possible due to the single event.

## Discussion

In efforts to expedite the exclusion of ACS in patients presenting with chest pain, we updated our clinical pathway to include a rapid discharge component for low-risk patients without the need for a second troponin. The data in this study showed that, in patients with HEART score ≤3 and undetectable initial troponin measurement, there were zero incidence of ACS and a minimal MACE at 30 days. However, despite an updated institutional protocol based on evidence supporting the safety of discharging patients based on a single undetectable troponin level, there remains notable hesitancy among clinicians to adopt this practice in our clinical setting.

Beyond clinical implications, this hesitancy amongst clinicians also has significant resource and cost considerations. Recent studies have shown that a significant portion of the cost burden associated with the evaluation of chest pain is attributable to low-risk patients with non-specific symptoms ^17^. As our data suggests, if providers were to carry out a single troponin discharge in these low-risk patients, this could substantially reduce retesting rates, ED LOS and, consequently, healthcare costs. While the median reduction in time amongst those who would qualify for a rapid discharge pathway is ∼1 h per patient, with the volume of chest pain patients seen in our ED annually, this translates to over 600 h (∼20-30 additional days) of ED access every year. The direct and indirect costs saved by the hospital could be astounding. These findings reinforce the need for ongoing implementation strategies to optimize resource utilization while maintaining patient safety.

Our data adds to the abundance of studies that a single high-sensitivity cardiac troponin assay below the LOQ can consistently rule out ACS in ED populations. A baseline high sensitivity troponin below the LOQ rules out ACS with a high degree of specificity and a negative predictive value 98%^4,5^. Based on this, Khand et al. recalibrated the HEART score to define time 0 as a high sensitivity troponin value less than the LOQ, and found that it increased the sensitivity of ACS rule-out using the HEART score at the cost of decreasing specificity^18^. Furthermore, Shah et al. evaluated patients with suspected ACS and found that a high sensitivity troponin below the LOQ retained prognostic value even up to 1 year with lower rates of adverse cardiac events compared to those with detectable troponin levels^19^.

The 2023 updates to European Society of Cardiology guidelines continue to support discharging low risk patients after a single high sensitivity troponin below the LOQ, citing both safety and cost-effectiveness^10^. Our early discharge pathway further reinforces this approach, demonstrating the safety of discharging low-risk patients using a combination of a HEART score ≤3 and a high-sensitivity troponin below the LOQ. However, it is important to note that several high sensitivity troponins exist on the market currently with varying LOQ, sensitivities and specificities. Only the Roche Diagnostics hs-cTn was used in our study.

Despite abundance of evidence of supporting single troponin discharge, studies examining actual clinician adoption of this practice is limited. Mahler et al. reported a non-adherence rate of approximately 20%, primarily due to over testing ^20^. Whereas in our study, we found that only about 50% of patient cases adhered to the single-troponin discharge protocol. This hesitancy may be attributed to a lack of trust in the updated algorithm and insufficient education. This finding also aligns with previous study by Marjot et al. ^21^, which have consistently demonstrated that, despite efforts to promote early discharge, a significant proportion of providers remain hesitant.

The rationale for this is likely multi-factorial. Insufficient education, systemic barriers, patient or caregiver level hesitancy, operational workflows, competing concerns (i.e., legal concerns), and staffing availability may all be contributing factors. Systematic, implementation-science guided strategies are needed to improve short-term compliance. Furthermore, long-term compliance was not assessed in this study. More long term data after additional quality improvement interventions may be beneficial. Potential barriers to new pathway implementation in the emergency department and potential strategies to overcome them are summarized in the Table 4.

**Table 4:** Barriers to implementing new diagnostic and discharge pathways in the emergency department and potential strategies to overcome them.

**Implementation Barrier**	**Potential strategy**
Limited knowledge in updated literature	Offer targeted in-person or virtual educational sessions for all clinicians to improve understanding of latest chest pain evaluation data and guidelines.
Lack of familiarity with updated protocol	Email reminders, question and answer sessions and educators on shift can help clinicians improve access and improve understanding of the updated protocol.
Resistance to change current practices	Protocol development leaders need to conduct multidisciplinary meetings to demonstrate pathway benefits through pilot studies after implementation.
EMR documentation limitations	Collaborate with EMR developers to integrate prompts or “hard stops” to document critical items such as HEART score
Disruption to current workflows	Automated EMR order sets based on initial troponin and documented HEART score may assist nursing workflow. Consultants and additional staff involvement in workflow also need training on protocol updates.

**Notes.**

EMR electronic medical record

## Limitations

This study has several limitations. As a single center study, conducted at a large, academic tertiary care facility, the findings are limited in their interpretation and generalizability to broader populations and other clinical settings. There is potential for unrecognized clinical biases during patient visit could have influenced protocol non-adherence amongst providers that were unable to be assessed in our retrospective study design. By focusing exclusively on low-risk chest pain patients with a HEART score of ≤3, the study included only a small subset of the initial cohort, introducing the possibility of selection bias. For example, patients that were already planned for admission may have been more likely to have a second troponin drawn. Or patients that appeared “ill” may be more likely to have a second troponin than another patient that is “well-appearing”. Additionally, more low risk features may have been documented in those that clinician wanted earlier discharge. Due to the minimal documentation of HEART scores in the EMR, our study only used retroactively calculated HEART scores which may not fully align with risk level determined clinically at the time of patient evaluation. Finally, the subjective nature of the “history” component of HEART score, which relies on chart reviewer interpretation, may compromise inter-rater reliability.

## Future Directions

Future prospective studies with larger sample sizes based on *a priori* sample sizes are needed to further investigate the validity of our results. Studies could explore the safety of a single undetectable troponin measurement in all chest pain patients, not just those classified as low risk. Additionally, further research could assess the necessity of a second troponin measurement for patients with initial troponin levels within the 99th percentile cutoff. It would also be pertinent to evaluate single troponins for other indications. For instance, a recent study by Leafloor^22^, suggests that a single troponin may also be sufficient for syncope evaluation. To validate these findings, prospective, randomized trials or larger cohort studies will be essential.

## Author contributions

Conceptualization: ZZ, JS, JK, JD, SS, VG,

Data collection: JS, AH, WM, DS

Data analysis: ES, KH

Writing –Draft preparation: ZZ, JS

Writing –Review and editing: ZZ, JS, ES, KH, AS, WM, DS, JK, DM, SS, VG

All authors have reviewed and approved final version of the manuscript

## Funding source

There was no funding for this study.

## Conflicts of interest

All authors report no conflicts of interest.

## Disclaimers

This research was supported in whole or in part by HCA Healthcare and/or an HCA Healthcare affiliated entity. The views expressed in this publication represent those of the authors and do not necessarily represent the official views of HCA Healthcare or any of its affiliated entities.

Large Language Models, e.g., ChatGPT, were used to make minor grammatical improvements in the text. Authors retain full responsibility for the scientific content, including data, analyses, and conclusions. All modifications facilitated by the tool were thoroughly reviewed and validated by the authors.
